# Commentary: Establishing the college Return to Learn team for concussion: a practical approach

**DOI:** 10.3389/fpubh.2023.1188741

**Published:** 2023-06-02

**Authors:** Zachary W. Bevilacqua, Jacob McPherson

**Affiliations:** ^1^Department of Exercise Science, Rochester Institute of Technology, Rochester, NY, United States; ^2^Department of Rehabilitation Sciences, University at Buffalo, Buffalo, NY, United States

**Keywords:** concussion, Return to Learn, college, higher education, policy, disabilities, FERPA, multidisciplinary team

## Introduction

Researchers have only recently begun to investigate Return to Learn (RTL) considerations among college students with concussion. Initial investigations were focused on academic reintegration timeframes (1), faculty and staff knowledge of concussion (2, 3), and impacts on academics (4). Since then, investigations have addressed more precise aims, namely faculty perspectives of RTL and classroom accommodations (5, 6), peer-mentoring programs for students recovering from concussion (7), the Athletic Trainers' role in RTL (8), and factors mediating reading performance (9, 10). Most recently, preliminary consensus recommendations were introduced (11), signifying that college RTL is distinct from K-12, and requires unique attention.

Consensus recommendations for college-RTL discuss routine check-ins with a multidisciplinary team (i.e., academic counselor, physician, instructor, and student-advocate) (11). Multidisciplinary teams have become a cornerstone of concussion management, yet a multidisciplinary approach in the college setting inherently encounters barriers, which have yet to be focally addressed in the literature. Specifically, the Family Educational Rights and Privacy Act (FERPA) disallows open communication between medical and academic entities on campus (12); the core characteristic of a RTL partnership. In turn, the onus of maintaining interdisciplinary communication is situated firmly with the student. To be successful, a RTL team must overcome such barriers if they hope to stay informed and properly chaperone students through a RTL protocol. To that end, we will discuss a reasonable approach to establishing communication between college-RTL team members (i.e., medical provider, disability services, faculty, etc.) in order to efficiently facilitate a student's RTL progress.

## Protocol

Literature discusses RTL as both the reintroduction of academic work post-concussion, and a state of recovery. Our protocol will refer to RTL in the former, as we do not define academic recovery, but instead outline RTL proceedings until such a time when they are no longer required. The following approach is comprised of three stages: receiving the diagnosis, activating the team, and follow-up.

### Receiving the diagnosis

**Figure 1:**
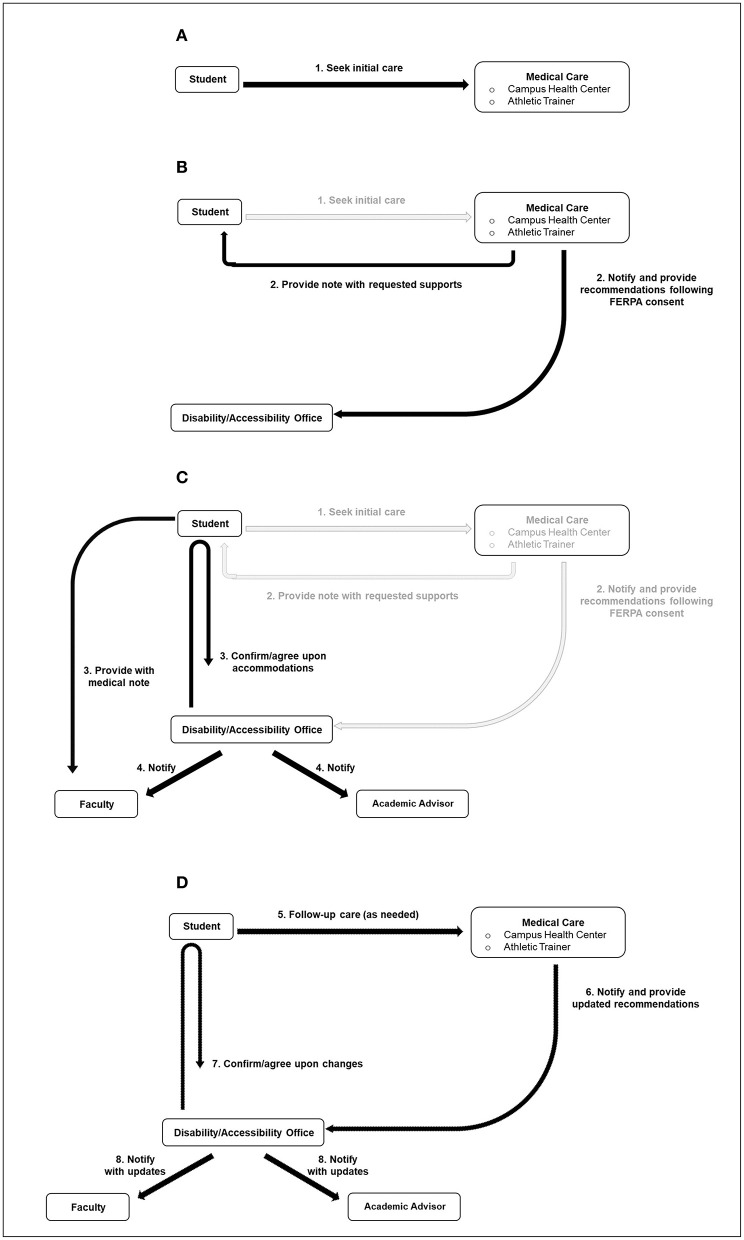
RTL team schematic. **(A)** Receiving the diagnosis. **(B, C)** Activating the team. **(D)** Follow-up.

Previous literature suggests that symptoms of concussion may persist for longer durations (i.e., >14 days) among student-athletes who receive fewer medical check-ins (13). Factors such as a pre-injury anxiety/mood symptoms (14), symptom severity (15), and sex (16) alter symptom resolution and time away from the classroom as well. If left unsupervised, the effects of such variables may have damaging repercussions on a student's semester and academic progress, especially when considering the pace of learning expected at the collegiate level. College-RTL already demands a mean 18.3 days when under medical care (1), with females requiring longer than males (16). Overall, 26% of college students will require more than 14 days to RTL (*n* = 1,974) (16), with up to 13% needing ≥35 days (1, 16). Therefore, to attenuate academic time lost to avoidable symptom prolongation, we encourage all students to seek care if a concussion is suspected. For college students, campus health centers are an excellent option, with Athletic Trainers representing the preferred route for student-athletes. If financial accessibility is a concern, the majority of college institutions offer health insurance plans to students that can be financed with financial aid packages. Additionally, the Affordable Care Act allows college-aged (i.e., <26 years old) individuals to remain insured through their parents (17). If barriers persist, Dean of Students Offices or other administrative entities on campus can provide assistance to students seeking concussion care. Whatever the means, obtaining medical supervision is the priority, and represents the first point-of-contact for students with concussion (Figure 1A).

### Activating the team

**Figure 2:**
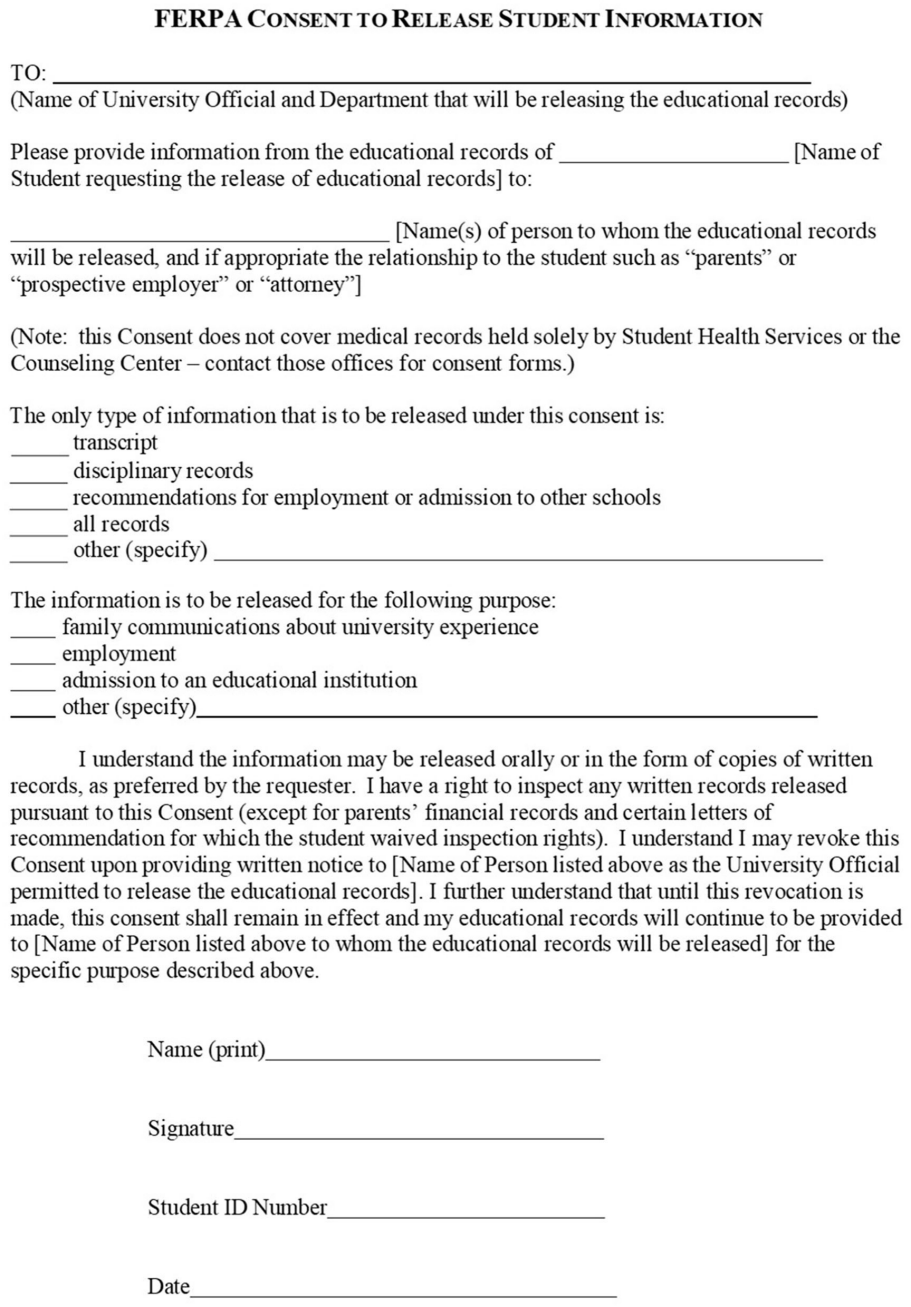
Sample FERPA consent form. Adapted from the Office of Legal Affairs at the Rochester Institute of Technology, 2023, https://www.rit.edu/fa/legalaffairs, Copyright Rochester Institute of Technology.

Medical evaluation provides obvious benefits, but it simultaneously triggers the next step of the RTL process (Figure 1B). Upon diagnosis, providers can offer students the option to sign a FERPA consent form (Figure 2) permitting them with the authority to share a student's diagnosis and treatment plan with the campus' disability office, or similar entity. Currently, the autonomous nature of the college environment often leaves students responsible for disseminating this information on their own, and is the product of FERPA restrictions (12); however, obtaining written approval is quickly accomplished in clinic, and will immediately permit interdisciplinary communication while alleviating students of this burden. FERPA consent could even merge into pre-participation paperwork for student-athletes, affording ongoing permission to Athletic Trainers and other members of the healthcare team. Likewise, initiation of cognitive reintegration is recommended no later than 48 h post-injury (18), so speed with communication is important. Lastly, it is speculated that campus disability offices lack the ability to assist students in a timely manner (11); therefore, integrating this form into clinical practice will allow team members to rapidly select classroom accommodations. In fact, dialogue between provider and disability staff could determine which accommodations will best support clinical findings (i.e., symptoms of photophobia managed by wearing sunglasses in class). Students pursuing accommodations throughout their recovery would likely find convenience in a protocol for authorizing open communication between the professionals responsible for implementing these supports. Therefore, we recommend that FERPA consent forms be considered as a routine tool used to facilitate rapid collaboration between “primary” RTL team members (campus health center and disability offices). Healthcare providers are also encouraged to provide these accommodation details to the student in the form of a discharge note (Figure 1B). This affords students the option to disseminate accommodation details to faculty (Figure 1C), should a student incur a delay in receiving disability assistance. Supplementary Figure 1 presents a sample note that has garnered favor from college faculty (19).

Students who have received medical care and are supported by disability staff would be able to expect “secondary” members of the RTL team to receive notification (Figure 1C). Faculty and academic advisors comprise this group, bearing a supportive role (5). In this way, faculty are instructed to adhere to the prescribed classroom supports, whereas academic advisors are apprised to the student's temporary struggle.

### Follow-up

Because students may receive follow-up care, and are anticipated to shed academic restrictions and accommodations as they progress through a RTL progression, routine communication will allow all to remain informed to any changes, and or when provided supports can be discontinued. Figure 1D illustrates this process.

## Rationale for the progression

The present protocol emphasizes receiving medical evaluation and the acquisition of campus disability support for two reasons. First, encouraging all students to receive medical attention follows recommendations to evaluate suspected concussion injury (18), and two, it produces a formal record of the injury to which campus disability offices use to justify “formal” accommodations (i.e., classroom supports that are justified by documentation) (20). Others would oppose our approach, stating that “informal” adjustments (i.e., classroom supports that are *not* supported by documentation) should be implemented quickly, and through student-faculty collaboration (11). We appreciate that a subset of students could secure informal adjustments through dialog with empathetic faculty (5); yet, this does not appear to be the majority of instructors (5, 6), nor is it assured that these informal adjustments will be entertained throughout the student's entire recovery. Furthermore, a growing body of literature indicates that faculty support is conditional (5–8), and requires disability and or medical documentation prior to implementing the supports that are routinely prescribed post-concussion (i.e., excused absences, extended time on tests/assignments, altered due dates, etc.) (5, 6, 19). In fact, nearly two-thirds (62.4%) of sampled faculty hold this opinion (*n* = 255) (6). Therefore, and in an effort to ensure equity with academic support, our approach urges the utilization of disability offices to guarantee formal accommodations in and outside of the classroom, and abate any biases faculty may have toward students (21–23). While disability services take a range of time to implement services and most students will RTL within 2 weeks, seeking academic accommodations is worthwhile given the lack of effectiveness of informal adjustments and that some students will require support for more than 35 days. We further encourage students to present instructors with their medical discharge note (Figure 1C), which half (48.6%) of faculty require (6), and respect similarly to disability notes (5). This could formally initiate accommodations prior to disability services reaching out to faculty (Figure 1C), and simultaneously provide an avenue for students who forego FERPA consent or disability offices altogether. Overall, efforts should focus on expediting the provision of formal accommodations, and promote education for students aimed at seeking concussion care. Should a student's recovery not follow a “typical” timeframe, medical records at the time of injury can be used to award a “medical withdrawal” or “incomplete” to a student. Generally speaking, the former allows a student to withdraw from the semester while recuperating a portion of their tuition dollars, while the latter provides the student the ability to complete course requirements the following semester. Both options are expedited by proof of medical necessity, and function as a safeguard of sorts.

Subsequent stages of the protocol sought to capitalize on known university procedures to establish a “point-person” and create channels of communication. A point-person has long been discussed as a critical component in a return-to-school effort for pediatric models, with the school nurse often identified for this role (24–26). RTL for college students will inherently look different, as universities do not have a single healthcare professional through which all medical information funnels (i.e., school nurse). Instead, disability offices are perhaps the ideal choice to assume point-person duties, as they currently interact with a student's medical and academic information, and are trained in producing academic orders from medical input. Additionally, they possess the infrastructure to electronically connect with academic team members, including students who are lost to follow-up. They also provide services that can directly assist faculty, such as administering examinations to a concussed student who requires additional time (27, 28).

## Conclusion

The present manuscript discusses a reasonable approach to establishing a RTL team in the college setting. The suggested plan discusses how medical evaluation and FERPA consent can kick start a RTL procedure, unencumbered. RTL authors cite that implementing a RTL protocol could take a mean 17 years if barriers and contextual factors are not addressed beforehand (29, 30). The suggested approach minimizes some of these concerns, utilizing campus infrastructure (health centers, athletic trainers, and disability offices) and processes (disability accommodations and FERPA consent) that have long been entrenched in higher education. Additionally, the medical records that result from the initial evaluation provide a multitude of uses, from justifying disability accommodations, to ameliorating faculty concerns, to safeguarding a student's investment in the semester. Overall, we encourage stakeholders (i.e., National Collegiate Athletic Association, National Athletic Trainers' Association, consensus protocols) to consider the practical and high-yield characteristics of the proposed procedure.

## Author contributions

ZB was responsible for the inception and drafting of the manuscript. ZB and JM contributed to the editing and refinement of the manuscript. Both authors contributed to the article and approved the submitted version.
