# Supplementary figures and images for: Commentary: Establishing the college Return to Learn team for concussion: a practical approach
Source: Front Public Health. 2023 Jun 2;11:1188741. doi: 10.3389/fpubh.2023.1188741 (PMC10306098; doi:10.3389/fpubh.2023.1188741)

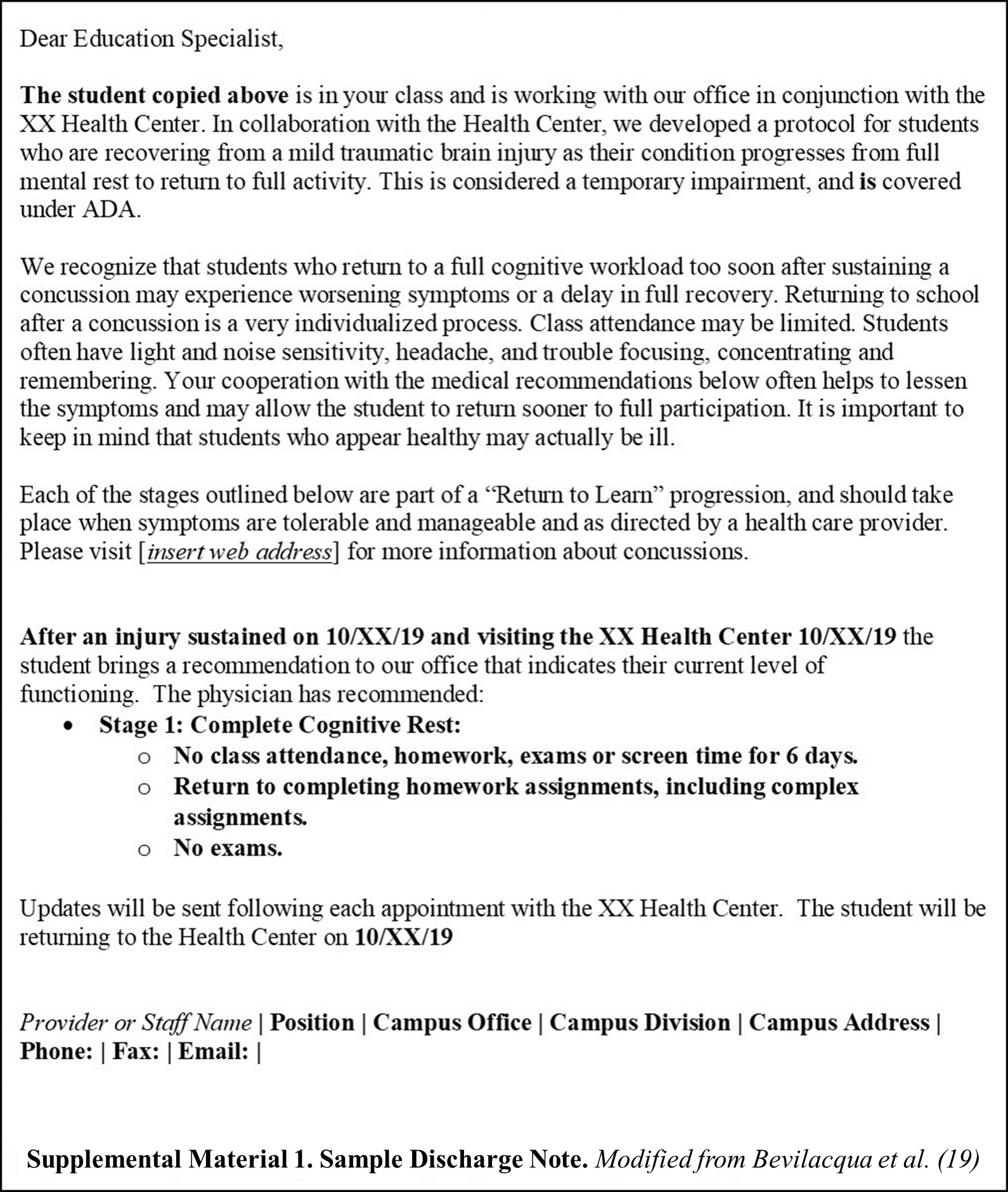

Supplement: Supplementary file 1 [file Image_1.JPEG]
